# Inactivated Orf Virus Shows Antifibrotic Activity and Inhibits Human Hepatitis B Virus (HBV) and Hepatitis C Virus (HCV) Replication in Preclinical Models

**DOI:** 10.1371/journal.pone.0074605

**Published:** 2013-09-16

**Authors:** Daniela Paulsen, Andreas Urban, Andreas Knorr, Claudia Hirth-Dietrich, Angela Siegling, Hans-Dieter Volk, Andrew A. Mercer, Andreas Limmer, Beatrix Schumak, Percy Knolle, Helga Ruebsamen-Schaeff, Olaf Weber

**Affiliations:** 1 AiCuris GmbH & Co.KG, Wuppertal, Germany; 2 Bayer HealthCare AG, Leverkusen, Germany; 3 Institute of Medical Immunology and Berlin-Brandenburg Center for Regenerative Therapies (BCRT), Charité – Medical University Berlin, Berlin, Germany; 4 Department of Microbiology and Immunology, University of Otago, Dunedin, New Zealand; 5 Institutes of Molecular Medicine and Experimental Immunology, University Hospital Bonn, Bonn, Germany; 6 Institute of Medical Microbiology, Immunology and Parasitology (IMMIP), University of Bonn, Bonn, Germany; 7 Institute of Molecular Immunology, Klinikum rechts der Isar, Technical University Munich, Munich, Germany; 8 University of Heidelberg, Heidelberg, Germany; Centro de Investigación en Medicina Aplicada (CIMA), Spain

## Abstract

Inactivated orf virus (iORFV), strain D1701, is a potent immune modulator in various animal species. We recently demonstrated that iORFV induces strong antiviral activity in animal models of acute and chronic viral infections. In addition, we found D1701-mediated antifibrotic effects in different rat models of liver fibrosis. In the present study, we compare iORFV derived from two different strains of ORFV, D1701 and NZ2, respectively, with respect to their antifibrotic potential as well as their potential to induce an antiviral response controlling infections with the hepatotropic pathogens hepatitis C virus (HCV) and hepatitis B virus (HBV). Both strains of ORFV showed anti-viral activity against HCV *in vitro* and against HBV in a transgenic mouse model without signs of necro-inflammation *in vivo*. Our experiments suggest that the absence of liver damage is potentially mediated by iORFV-induced downregulation of antigen cross-presentation in liver sinus endothelial cells. Furthermore, both strains showed significant anti-fibrotic activity in rat models of liver fibrosis. iORFV strain NZ2 appeared more potent compared to strain D1701 with respect to both its antiviral and antifibrotic activity on the basis of dosages estimated by titration of active virus. These results show a potential therapeutic approach against two important human liver pathogens HBV and HCV that independently addresses concomitant liver fibrosis. Further studies are required to characterize the details of the mechanisms involved in this novel therapeutic principle.

## Introduction

Hepatitis C virus (HCV) infection and infection with human hepatitis B virus (HBV) remain major medical problems. There are an estimated 130 million people chronically infected with HCV with 350,000 of them dying due to HCV-related liver disease [1]. In addition, there are approximately 350 million people chronically infected with HBV and 65 million of those have a high risk of dying from liver disease due to their HBV infection [2]. Therefore, chronic HCV as well as HBV infection is considered a major risk of cirrhosis, hepatocellular carcinoma (HCC) and liver failure [3], [4]. Unlike the situation in HBV, a protective HCV vaccine is not available and, despite existing therapies, the number of patients presenting with chronic HCV infection and resulting complications such as HCC may further increase mid-term [3]–[5].

Interferon α (IFN-α) has been an integral part of HCV-specific therapies with telaprevir- or boceprevir-based triple combinations with pegylated (peg)IFN-α and ribavirin becoming the novel standard therapy for HCV genotype I patients [6], [7]. Despite the availability of HCV-specific treatment options a significant medical need remains specifically for patients, where combination therapies with pegIFN-α or ribavirin are contraindicated, such as patients with decompensated liver cirrhosis or liver transplant failure [7]. Therefore, the need to provide novel and effective therapies for treating chronic hepatitis C remains high.

Orf virus (ORFV, *Parapoxvirus ovis*) is an epitheliotropic DNA virus that belongs to the genus *Parapoxvirus* of the *Poxviridae* family. ORFV causes an acute skin disease in sheep and goats worldwide [8]. Several putative immune-modulating ORFV genes have been described including a viral orthologue of IL-10 [9], [10].

We recently demonstrated that inactivated ORFV (iORFV) strain D1701 induces a systemic activation of the immune system and strong antiviral activity in animal models including HBV–transgenic mice [11]. We also showed that IFN-γ was a key mediator of the iORFV-mediated antiviral activity as the neutralization of IFN-γ activity led to a marked reduction of the antiviral effects *in vivo* [11]. Despite the strong iORFV-mediated antiviral activity in the HBV-transgenic mouse model, we did not observe histological or clinical signs of inflammation. We furthermore described the antifibrotic activity of iORFV, strain D1701 in rat models of liver fibrosis after induction by carbon tetrachloride (CCL_4_) or pig serum administration [12]. Because IFN-α is among the cytokines induced by iORFV [13] we now attempted to study potential anti-HCV effects of iORFV in a cell-based model system for HCV replication [14]–[16]. Moreover, we compared iORFV derived from two different strains of ORFV, D1701 and NZ2, respectively, to evaluate their antifibrotic potential as well as their potential to inhibit HBV as well as HCV replication.

Both strains of ORFV showed anti-viral activity against HCV *in vitro* and against HBV in a transgenic mouse model without signs of necro-inflammation *in vivo*. Our experiments suggest that the absence of liver damage is potentially mediated by iORFV-induced downregulation of antigen cross-presentation in liver sinus endothelial cells. Furthermore, both strains showed significant anti-fibrotic activity in two rat models of liver fibrosis. However, iORFV strain NZ2 appeared more potent compared to strain D1701 in both its antiviral and antifibrotic activity based on dosage calculations using tissue culture infectious dose 50 (TCID_50_) of active virus.

## Materials and Methods

### Virus

*Parapoxvirus ovis* (ORFV) strain NZ2 or D1701 was propagated in bovine kidney cells (BK KL3A) isolated from a fetal calf in 1963 [17]. Briefly, the virus was harvested when approximately 80–90% of the cells showed a cytopathic effect. Debris was removed by centrifugation. The supernatant was decanted and centrifuged (Beckman, SW 28) for 6 hours at 20.000 rpm, 4°C. Subsequently, the pellet was resuspended and titrated in a plaque assay on BK KL3A cells. The virus was purified through a sucrose gradient and inactivated using binary ethylenimine (BEI). Successful inactivation was confirmed by re-titration of virus on BK KL3A cells. Inactivated ORFV (iORFV) was suspended in phosphate-buffered saline (PBS) and dosed as indicated.

### Cytokine mRNA Measurement

Total RNA was prepared according to the method of Chromczynski and Sacchi [18] and gene expression of IFN-γ was quantified using a competitive RT-PCR as described previously [11]. Briefly, female BALB/cJ mice (4 weeks old, approximately 20 g body weight) were purchased from a commercial supplier (Bomholdtgard). iORFV was administered at various dosages, tissue-culture infective dose 50 (TCID_50_) was estimated by titration of active virus. Mice were divided into three treatment groups (n = 12 animals per group): (i) iORFV strain D1701 (5×10^4^ TCID_50_), or (ii) iORFV strain NZ2 (5×10^4^ TCID_50_) both diluted in 200 µl non-pyrogenic phosphate-buffered saline (PBS, Seromed); and (iii) non-pyrogenic PBS (placebo). At 6 or 12 h after treatment, six mice/group were sacrificed and peritoneal cells were collected and analyzed for IFN-γ expression. Experimental results have been confirmed in independent experiments.

### Test of iORFV for Inhibition of HCV Replication

To determine the anti-HCV activity of iORFV, the stable HCV replicon cell lines Huh5-2 [15] and a NS3-based HCV FRET assay [16] were used. In a first step, blood from a healthy human donor was obtained using a S-Monovetten Lithium-Heparin (Sarstedt, Nuembrecht, Germany) and incubated for three days with PBS, Concanavalin A (ConA) or iORFV, strain NZ2 or D1701, diluted 1∶5 in complete DMEM (LifeTechnologies, Darmstadt, Germany) at 37°C. Dosages of iORFV were determined by TCID_50_ of active virus as described above and doses of approx. 8×10^6^ TCID_50_ have been used for exposure of human blood with iORFV from both strain D1701 or NZ2, respectively. After three days the blood cells were pelleted (230 g, 5min) and 15 µl of the supernatant was transferred onto Huh5-2 cells, which were seeded at a density of 6×10^3^ cells per well in a 96-well plate in complete DMEM supplemented with 10% heat-inactivated fetal calf serum (Biochrom, Berlin, Germany), 1×MEM non-essential amino acids solution (LifeTechnologies, Darmstadt, Germany) and 250 µg/ml G418 at 37°C and 8.5% CO_2_. Human IFN-α (I 4276, Sigma, Taufkirchen, Germany) at various concentrations ranging from 250 to 0.0075 units/ml was used to confirm the functionality of the system and the effective concentration 50 (EC_50_) of 3.3 units/ml determined in our experiments was in line with published information [16]. Huh-7 cells without the HCV replicon and untreated Huh5-2 cells were used as 0% and 100% signal control. All reaction wells had a total volume of 200 µl. In order to reduce edge effects, the 36 wells at the outer rim of the 96-well plate were not used, but were filled with assay medium. After three days cells were rinsed with phosphate buffered saline and NS3 protease activity as an accurate readout for HCV replication was determined by using a FRET assay which has been described previously [16]. Reproducibility of the iORFV-mediated anti-HCV activity was confirmed in independent experiments.

### Animals

- HBV transgenic mice: Male HBV transgenic mice [Tg (HBV1.3 fsX-3′5′)] were used (n = 7 per treatment group) [11], [19]. The mice used in the experiments produced 10^7^–10^8^ HBV genome equivalents per ml plasma as determined by quantitative real time PCR [18].
- For induction of liver fibrosis with either pig serum or Carbon tetrachloride (CCl_4_) in rats, female Sprague Dawley rats at a body weight of 220–240 g (Moellegaard, Lille Skensved, Denmark) were used.

Studies were approved by the competent authority for labor protection, occupational health and technical safety and performed in accordance with the ethical guidelines of Bayer HealthCare Pharmaceuticals as well as the guidelines of the local Laboratory Animals Science & Welfare Council. Studies were carried out in strict accordance with German law (“Tierschutzgesetz”) and the European guidelines (86/609). The work was approved by the Regierungspräsidium Düsseldorf Karl-Rudolf-Str.180 in 40408 Düsseldorf (Permit Number: AZ23.05-240-1-76/96). All efforts were made to minimize suffering.

### Treatment of HBV-transgenic Mice and Analysis of HBV-specific DNA or Antigen

iORFV strain NZ2 or iORFV strain D1701 was administered to HBV transgenic mice [Tg (HBV1.3 fsX-3′5′)] i.p. on days 1 and 4, two times in total, at doses of 1.5×10^6^ TCID_50_, 5×10^5^ TCID_50_, 1.5×10^5^ TCID_50_ and 5×10^4^ TCID_50_. Animals were sacrificed on day 5 and plasma and livers were subjected to HBV-DNA analysis. Quantitative analysis of hepadnaviral nucleic acid in livers of HBV-transgenic mice was performed using dot-blot analysis and HBV DNA in plasma was analyzed by quantitative PCR essentially as described previously [11], [19]. Immunohistological analyses of liver specimen using a 1∶500 diluted polyclonal rabbit antibody against HBcAg (Dako, Hamburg, Germany) were performed essentially as described [11], [19]. In addition, n = 3 HBV-transgenic mice were treated with oligonnucleotide ODN 1668 (5′-tccatgacgttcctgatgct-3′) [20]. ODN was dissolved in 200 µl PBS and administered at a dosage of 50 µg/mouse intravenously. The study on anti-HBV activity of iORFV was repeated to ensure reproducibility. However, the full dose response in HBV-transgenic mice did include three instead of four dosages in the repeat although in individual experiments each dose has been repeatedly studied. Furthermore, inclusion of n = 7 mice/group supports robustness of the findings.

### Pig Serum-induced Liver Fibrosis

ORFV prevents both the development of fibrosis and the activation of collagen-producing cells in rat models of liver fibrosis [12], [21]. iORFV strain NZ2 was administered intraperitoneally at doses of 1.5×10^5^ TCID_50_/rat or 5×10^5^ TCID_50_/rat three times per week concomitantly to the fibrotic stimulus (porcine serum). Rats (3 groups with n = 15/group) received 0.5 ml porcine serum, P 9783 (Sigma Aldrich, Germany) intraperitoneally (i.p.) two times per week. Non-fibrotic control rats (n = 10) received PBS i.p. iORFV NZ2 was administered at doses of 1.5×10^5^ TCID_50_ and 5×10^5^ TCID_50_ or PBS i.p. three times per week concomitantly to the porcine serum or PBS treatment for seven weeks. Rats were sacrificed after 7 weeks and livers were subjected to collagen determination. Serial sections were stained with Sirius Red (fibrous collagen) and scored using a three class system (normal histology, beginning fibrosis with sprouting, unconnected septae and full-blown fibrosis with a network of interconnected septae). Hydroxyproline was measured as described recently (12). Picosirius stained scar collagen was quantified as described below for CCl_4_-induced liver fibrosis.

iORFV-mediated antifibrotic activity was confirmed in independent experiments. Furthermore, inclusion of n = 15 rats/group ensured the robustness of the findings.

### Carbon Tetrachloride (CCl_4_) - induced Liver Fibrosis

Induction of CCl_4_-mediated liver fibrosis was performed as described recently [12]. Briefly, rats received a single intragastric bolus of 1 ml 40% CCl_4_ in liquid paraffin. iORFV or placebo (PBS) were administered i.p. afterwards. After 48 hours, serial liver sections (3 µm) were stained immunohistologically for α-smooth muscle actin. Immunohistology was performed as described [12]. The percentage of a standardized centrilobular area stained was determined for each animal by an automated, blinded procedure. In order to facilitate comparison, the staining of liver sections obtained from animals treated with CCl_4_ and vehicle only was set as 100%. Histology and morphometric analysis was performed essentially as described recently [12]. Briefly, for morphometry of picosirius stained scar collagen we used a Leica QWin image analysis and processing system with motorized stage. For detection of smooth muscle actin (SMA) we used the monoclonal mouse anti-α−SMA antibody, clone 1A4 (Sigma Aldrich Germany). Reproducibility of the results has been confirmed in independent experiments.

### Cross Presentation Assay in Liver Sinus Endothelial Cells (LSEC)

Pure populations of LSEC were isolated from murine liver by a stepwise procedure of portal-vein perfusion with collagenase A (0.05%), mechanical dispersion and further enzymatic digestion in a rotatory waterbath for 40 minutes at 37°C (245 rpm), gradient centrifugation (metrizamide 1.089 g/cm3) and centrifugal elutriation using a Beckman Avanti J25I centrifuge equipped with a JE-6B rotor and a standard elutriation chamber. LSEC cell populations isolated by this method [22] were around 95–99% pure as measured by uptake of endothelial cell specific substrate (acetylated low density lipoprotein). LSEC were seeded onto collagen type I coated 24-well tissue culture plates at a density of 100.000 cells per well and were further cultured in DMEM supplemented with 5% fetal calf serum (specially tested not to interfere with the assay system) and 2% glutamine. Three days after isolation, when LSEC gained a post-mitotic and quiescent state, we tested for the ability of LSEC to cross-present soluble ovalbumin to (ovalbumin-specific) CD8+ T cells. LSEC were incubated with 1 µM of ovalbumin for three hours, washed and incubated with the CD8+ T cell hybridoma B3Z [23] (200.000 cells/well) that recognizes the peptide SIINFEKL presented on H2Kb MHC class I molecules (derived from ovalbumin following proteasomal processing and TAP-mediated loading onto MHC class I molecules in the ER). Either PBS (mock-treatment) or 5×10^5^ TCID_50_ iORFV strain D1701 were added. The extent of cross-presentation leading to CD8+ T cell activation was measured by determining the extent of IL-2 release from T cells into the cell culture supernatant by specific sandwich ELISA (BD Biosciences, Heidelberg, Germany). The experiment has been repeated to ensure reproducibility.

## Results

### iORFV-mediated Anti-HBV Activity in HBV-transgenic Mice

**Figure 1:**
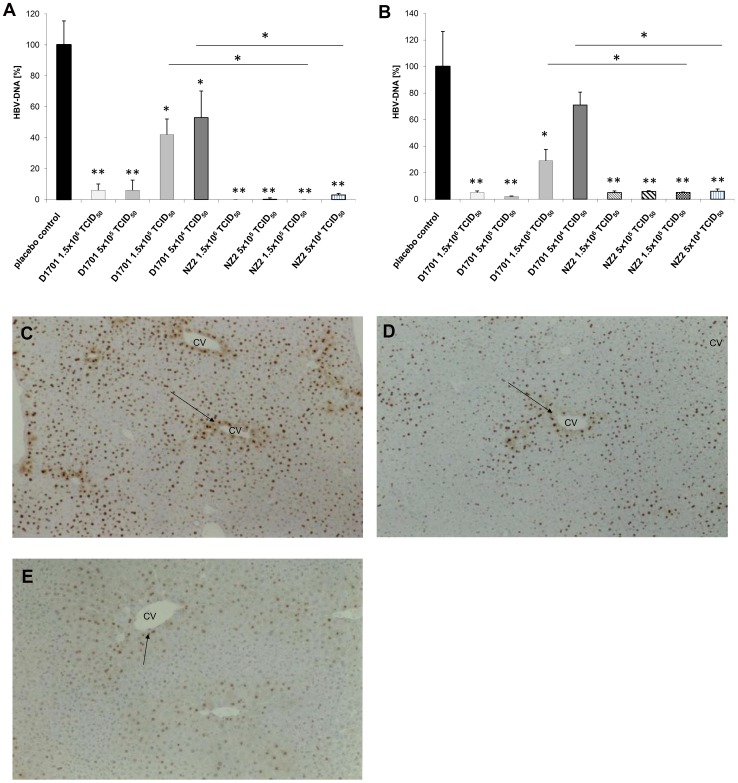
iORFV NZ2 is a more potent inhibitor of HBV than strain D1701 in HBV-transgenic mice. iORFV D1701, iORFV NZ2 or placebo was administered i.p. every third day, three times in total, to HBV-transgenic mice expressing 10^7^–10^8^ HBV genome equivalents per ml plasma (n = 7 per group). HBV-specific DNA from plasma was analyzed by quantitative PCR and from livers by dot-blot hybridization as described previously [11], [18]. The figure shows means ± standard error of means (SEM) relative to placebo-treated animals where mean HBV-DNA content was set 100%. Treatment with iORFV reduced non-chromosomal HBV-DNA in the livers as compared to placebo animals **(a)** and in the plasma with the exception of the lowest dose of iORFV D1701 **(b)**. iORFV NZ-2 was more potent in terms of its potential to inhibit HBV replication compared to strain D1701 with the two lower dosages resulting in significantly higher reduction of HBV-DNA (one-way ANOVA and *post hoc* analysis [Newman-Keuls Multiple Comparison Test] *p<0.05, **p<0.01). **c)** Immunohistological analysis of HBcAg expression in the livers of placebo-treated animals. Diffuse cytoplasmic staining in periportal areas [arrows, central veins (CV)] indicates viral capsids and ongoing HBV replication in placebo-treated mice. Cytoplasmic HBcAg as well as nuclear HBcAg-specific stain (for empty capsids) was strongly reduced in both iORFV (NZ2)-treated **d)** and iORFV (D1701)-treated mice **e)**. Figures provide typical examples of the respective group of animals treated with a dose of 1.5×10^6^ TCID_50_ iORFV NZ 2 or D1701, respectively.

iORFV treatment potently inhibited HBV replication in the HBV-transgenic mouse. In livers of HBV-transgenic mice treated with iORFV D1701 non-chromosomal HBV-DNA was reduced by more than 90% compared to livers of placebo-treated mice at the two high doses and approximately 50–60% less HBV DNA as compared to placebo treatment was detected at the two lower dosages of iORFV. iORFV NZ2 almost completely reduced HBV-DNA at all doses applied (figure 1a). Various doses of both iORFV strains, but not the lowest dose of iORFV strain D1701 significantly reduced HBV DNA in the plasma (figure 1b). Liver tissue specimens were subjected to immunohistological analyses in order to assess the influence of iORFV application to HBV core antigen (HBcAg) expression in hepatocytes. In placebo-treated control animals HBcAg was detected in the hepatocyte cell nuclei and the cytoplasm of centrilobular hepatocytes (figure 1c). Cytoplasmic HBcAg was markedly reduced after administration of the two highest dosages of iORFV D1701 (figure 1d) and the reduction was even more pronounced after administration of iORFV NZ2 (figure 1e). Treatment with iORFV also reduced nuclear HBcAg-specific staining. The extent of HBcAG-specific staining was quantified according to a scoring from 1 to 3 (table 1). Briefly, the reduction of HBcAg-positive hepatocytes was more pronounced in the NZ2-treated mice compared to those treated with iORFV D1701. In addition, cytoplasmic HBcAg was undetectable in hepatocytes throughout the liver in n = 16 out of n = 28 animals treated with iORFV NZ2. There was no histological evidence for a necrosis or an inflammatory reaction in livers of mice treated with iORFV. In addition, pathohistological analysis did not reveal other pathological findings.

**Table 1:** Treatment with inactivated ORFV reduces HBV core antigen (HBcAg) in HBV-transgenic mice (n = 7/group).

Treatment	placebo	iORFV D1701				iORFV NZ2			
Dose [TCID_50_]	–	1.5×10^6^	5×10^5^	1.5×10^5^	5×10^4^	1.5×10^6^	5×10^5^	1.5×10^5^	5×10^4^
Mean Anti-HBcAg severity score: Nuclei	3.0	2.7	2.1	2.7	3.0	3.0	2.7	2.7	2.3
Mean Anti-HBcAgseverity score:Cytoplasm	2.9	1.5	1.7	2.7	2.4	1.8	1.0	2.0	1.8

* Scores of anti-HbcAg stain: Nuclei: grade 3: the majority of nuclei in the liver specimens revealed intense immunostaining; grade 2: the number of positive nuclei was slightly reduced predominantly at the periphery of the liver lobule; grade 1: further reduction of positive nuclei was observed. Cytoplasm: grade 3: strong staining of numerous hepatocytes was observed around the central veins; grade 2: the cytoplasmic staining was reduced; grade 1: a further reduction or even absence of cytoplasmic immunostaining was observed. The histological slides were cross-checked by an independent pathologist and the results confirmed.

The severity of HBcAG-specific stain was quantified according to a scoring from 1 to 3*.

### iORFV-induced Anti-HCV Activity In-vitro

**Figure 2:**
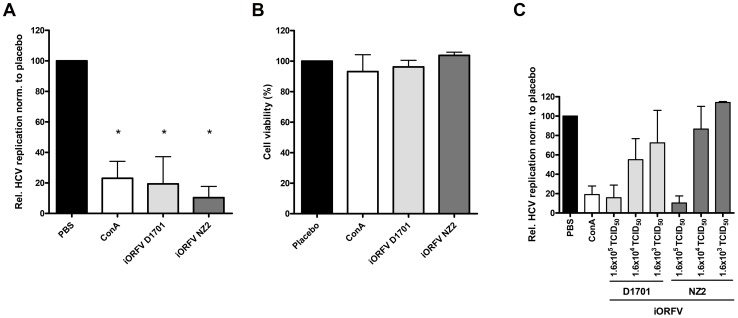
Both ORFV strains induce cytokines suppressing HCV replication in an *in vitro* replicon system. Figures shows relative fluorescence units (RFU) as a measure for HCV replication. **a)** 20 µl (dose 1.6×10^5^ TCID_50_) iORFV strain D1701 or NZ2 was added to whole blood and incubated for 3 days (see methods). Supernatants of cultured blood cells were added to replicon cells and HCV replication was determined three days later. Statistical analyses were performed using the one-way ANOVA and as *post hoc* analysis the Bonferroni Test. *p<0.05 vs. placebo. RFU-value from placebo (PBS) - treated supernatants was set as 100%. ConA served as a positive control. **b)** Cell viability of replicon-bearing cells was not affected after addition of supernatants from iORFV-incubated human blood cells. **c)** iORFV inhibits HCV replication in a dose-dependent manner. D 1701 or NZ2 were used for incubation with whole blood at doses ranging from 1.6×10^5^ TCID_50_ to 1.6×10^3^ TCID_50_. Values (n = 3) show means ± standard error of means (SEM).

Incubation of HCV replicon cells with human plasma obtained from human blood cells that had been incubated with iORFV for 3 days resulted in significant inhibition of HCV replication (figure 2a). Viability of replicon-bearing cells was not affected as determined by alamar blue staining (figure 2b). Cell viability was also not influenced by human plasma from blood cells that had been mock treated or treated with Con A. Both strains of iORFV reduced HCV replication in a dose-dependent manner (figure 2c). Importantly, both the D1701 and the NZ-2 strain of iORFV induced strong anti-HCV activity. Thus, iORFV initiates a strong anti-viral response against HBV and HCV.

### iORFV Inhibits Antigen Cross-presentation in Liver Sinus Endothelial Cells (LSEC)

Administration of iORFV did not lead to an inflammatory reaction in the livers of HBV-transgenic mice despite the induction of several Th1-type cytokines. In contrast, CpG oligonucleotide -mediated anti-HBV activity in HBV-transgenic mice was accompanied by necroinflammation in the liver (not shown). LSEC cross-present exogenous ovalbumin very efficiently on MHC class I molecules (H2Kb) to CD8+ T cells. When LSEC were incubated with iORFV we observed a potent downregulation of antigen cross-presentation by approximately 80% compared to mock-treated LSEC (table 2). However, activation of T-cells was not negatively impacted by iORFV. Exposure of LSEC and T cells with the SIINFEKL antigen in presence of iORFV led to increased levels of IL-2 secretion indicating that cross-presenting capacity of antigen-presenting LSEC was modulated by iORFV. The cross presentation test was validated using the non-OVA-related beta-GAL peptide DAPIYTNV which did only result in background IL-2 levels of 34±4 pg/ml whereas ovalbumin incubation led to the production of 1460±140 pg/ml IL-2.

**Table 2:** iORFV (D1701) reduces antigen cross-presentation by liver sinus endothelial cells (LSEC) but enhances activation of T-cells.

antigen	LSEC+B3Z+PBS (mock-treated)	LSEC+B3Z+ iORFV	comparison *vs*. mock-treated LSEC (100%)
ovalbumin	1084 pg/ml IL2	236 pg/ml IL2	22%
SIINFEKL	1780 pg/ml IL2	3043 pg/ml IL2	168%

Activation of CD8+ T cells (B3Z) was measured by IL-2 secretion.

### Induction of IFN-γ Expression by iORFV, Strains D1701 and NZ2

**Figure 3:**
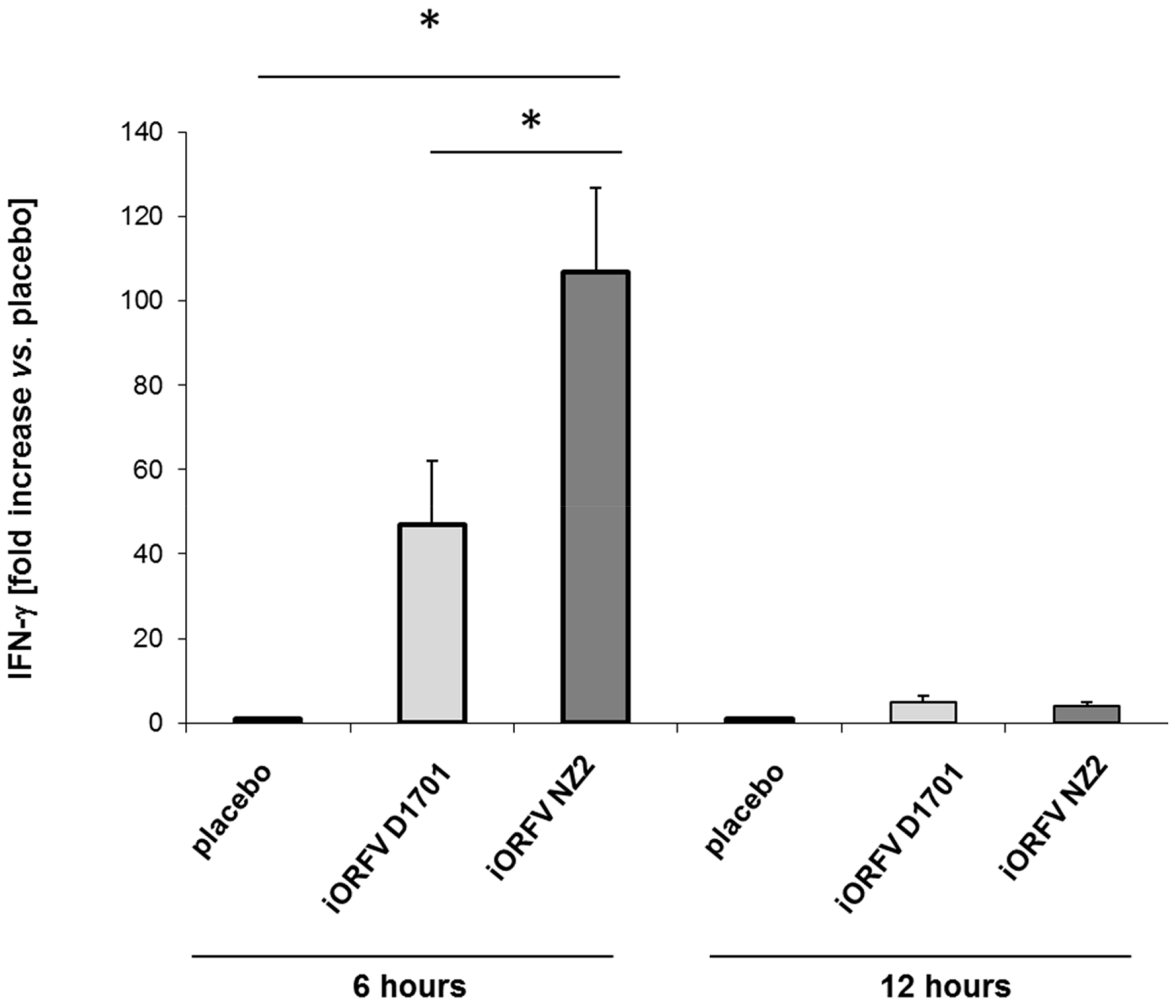
iORFV strain NZ2 is a more potent inducer of IFN-γ as compared to strain D1701. BALB/c mice (n = 6/group) were treated i. p. with iORFV at a dose of 5×10^4^ TCID_50_ of either strain of iORFV. PBS-inoculated animals served as control. Peritoneal lavage cells were collected 6 or 12 h after iORFV administration and analyzed for IFN-γ expression using a quantitative RT-PCR. Values shown are means ± standard error of means (SEM). Statistical analyses were performed using the one-way ANOVA and as *post hoc* analysis the Newman-Keuls Multiple Comparison Test. *p<0.05, ***p<0.001.

Peritoneal lavage cells have been a useful marker for overall IFN-γ expression in various organs and tissues after administration of iORFV [11]. Comparing the IFN-γ expression profile induced by iORFV strain NZ2 vs. strain D1701 revealed distinct differences in female 4 week-old BALB/cJ mice as displayed in figure 3. NZ2 induced significantly higher levels of IFN-γ expression 6 h after treatment compared to strain D1701. Twelve hours after treatment the mRNA levels returned to placebo levels with both virus strains.

### iORFV-mediated Activity in Pig Serum- induced Liver Fibrosis

**Figure 4:**
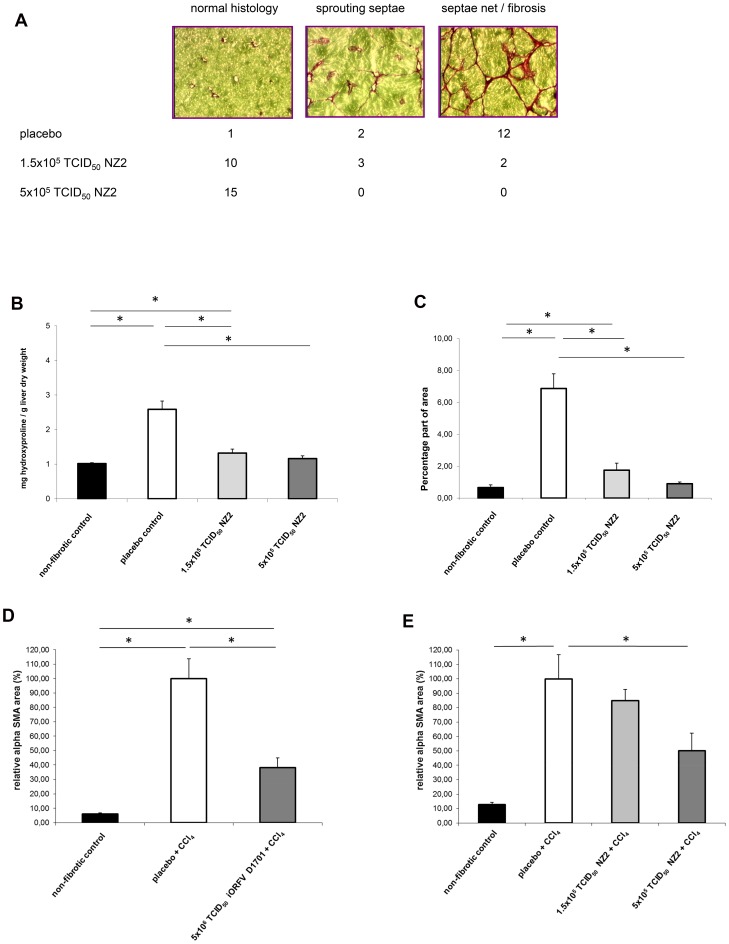
ORFV prevents fibrosis in a model of serum-induced liver fibrosis. iORFV strain NZ2 was administered intraperitoneally at doses of 1.5×10^5^ TCID_50_/rat or 5×10^5^ TCID_50_/rat three times per week concomitantly to the fibrotic stimulus (porcine serum). Rats were sacrificed after 7 weeks and livers were subjected to collagen determination. Serial sections were stained with Sirius Red (fibrous collagen) and scored using a three class system **a)** The figure shows histological sections of rat livers (magnification 25×). The three classes (normal histology, beginning fibrosis with sprouting, unconnected septae and full-blown fibrosis with a network of interconnected septae) are illustrated by histological pictures and the number of animals with this score is given below for the three experimental groups. **b)** Total collagen was quantified by determination of hydroxyproline. **c)** Fibrous collagen was quantified by measuring the percentage of area stained by Sirius red (see Material and Methods) in the histological sections. Both iORFV strain D1701 **d)** as well as NZ2 **e)** inhibit a key step in liver fibrogenesis, the transformation of fat-storing hepatic stellate cells onto myofibroblasts. HSC transformation was induced *in vivo* by a single gastric bolus of CCl4 as described in the method’s section. After 48 h the rats (n = 6/group) were sacrificed and liver myofibroblasts were detected immunohistologically by staining for α-smooth muscle actin (α-SMA). The stained area in a standardized centrilobular field was determined for each animal by an automatized, blinded procedure and expressed as the percentage of the corresponding mean value in placebo-treated rats **a)**. Parameters were analyzed using the unpaired t-test with Welch’s correction; all groups were analyzed using one-way ANOVA with *post hoc* Newman-Keuls Multiple Comparison Test.

In a next step we aimed to study the effect of iORFV strain NZ2 on liver fibrosis. Dose response studies with inactivated ORFV were performed using a model of pig serum–induced liver fibrosis in rats. This experimental model system is characterized by septal fibrosis in the absence of severe hepatic necro-inflammation (figure 4a) compared to healthy controls. Treatment with iORFV NZ2 dose-dependently prevented the formation of fibrotic septa. Quantitation of the effect of ORFV by determination of hydroxyprolin and morphometry of Sirius Red-stained fibrous collagen revealed a significant inhibition of hepatic fibrosis (figures 4b and c). The high ORFV dose (5×10^5^ TCID_50_) provided complete protection against liver fibrosis, administration of the low dose of 1.5×10^5^ TCID_50_ led to a significant reduction of fibrosis.

### iORFV-inhibits Transformation of Hepatic Stellate Cells (HSC)

In a model of early liver fibrosis fat-storing HSC transform into myofibroblasts. This transformation is accompanied by the expression of α-smooth muscle actin (α-SMA) by transformed cells. Our data show that iORFV inhibits transformation of HSC into α-SMA-expressing cells and that a dose of 5×10^5^ TCID_50_ of strain NZ2 and a dose of 5×10^6^ TCID_50_ of strain D1701 is sufficient to elicit an inhibitory effect on HSC transformation above 50% (figure 4d).

## Discussion

In this report, we show that strains of inactivated orf virus (iORFV) induce immune defense against important human liver pathogens, HBV and HCV, in preclinical model systems. In addition and independently of its antiviral efficacy, iORFV is able to prevent liver fibrosis, which is a major complication of chronic liver inflammation induced by chronic HCV or HBV infection [24], [25]. ORFV is known as a potent immune modulator in various animal species [8], [9], [10], [26] and induced the release of pro-inflammatory cytokines and also anti-inflammatory cytokines in human immune cells *in vitro* [27]. We recently demonstrated that iORFV D1701 induces strong antiviral activity in animal models of acute and chronic viral infections including a HBV-transgenic mouse [11]. As neutralization of IFN-γ with monoclonal antibodies abolished the antiviral effects in HBV-transgenic mice, we aimed to confirm the IFN-γ-inducing potential of a different strain of ORFV as an appropriate surrogate marker to investigate another strain of ORFV for its antiviral therapeutic potential [11]. Importantly, the antiviral activity was observed in absence of an inflammatory response regardless the ORFV strain used in the experiments. In contrast, CpG-oligonucleotide mediated anti-HBV activity was accompanied by necroinflammation in livers of HBV-transgenic mice. The absence of an inflammatory reaction in the livers of HBV-transgenic mice after administration of iORFV may be explained by iORFV-mediated downregulation of antigen cross-presentation by LSEC by approx. 80% compared to mock-treated LSEC as implied by differences in IL-2 productionafter interaction of ovalbumin-specific T cells incubated with ovalbumin and the peptide SIINFEKL [28]. Soluble ovalbumin, in contrast to the ovalbumin-derived peptide SIINFEKL, requires uptake and processing within the cell. The mechanism of cross-presentation involves uptake of protein into vesicles, export to the cytosol, digestion by the proteasome and reimport into vesicles by the peptide transporter TAP [29]. In contrast, peptides can directly bind to MHC molecules at the cell surface by exchanging already bound (lower affinity) peptides from MHC molecules. Although stimulation of LSEC with CpG oligonucleotides *in vitro* has not affected T cell tolerance induction *in vitro* [30] administration of CpG oligonucleotides caused necroinflammation *in vivo* [31]. This suggests the involvement of more than one type of liver cells. iORFV and CpG may induce different effects on Kupffer cells. Whereas CpG oligonucleotides - via binding to TLR9/MyD88 - strongly induce IL-6 and IL-12 (but no type I interferon) [32] iORFV has been shown to strongly induce type I interferons independently of MyD88 [33]. In addition, iORFV has been suggested to induce suppressive effects on macrophages [34]. While TNFα and IL-12 are known to cause liver damage [35], type I interferon has been shown to be liver protective [36]. We hypothesize that iORFV may protect HBV-expressing hepatocytes from immune attack by two mechanisms i) reducing LSEC cross-presentation of viral antigens to activated HBV-specific cytotoxic CD8+ T cells [37] and ii) by inducing liver-protective cytokines such as type I interferon [33] as well as IL-10 [11]. Use of CpG-oligonucleotides has been suggested to stimulate a broad spectrum of innate and subsequent adaptive immunity to combat chronic viral diseases or tumors. The absence of necroinflammation after administration of iORFV- but not after CpG-oligonucleotide administration - may be an important differentiator between iORFV and CpG-oligonucleotides.

ORFV strain NZ2 is one of the most well studied ORFV isolates [38]. NZ2 was originally isolated from sheep scab material and plaque purified twice in primary bovine testis cells [39]. The NZ2 genome is 138 kbp and contains 132 putative genes, each of which is also present in the genomes of 2 other fully sequenced isolates of ORFV, SA00 and IA82, that like NZ2 are representative of wild type ORFV [38]. ORFV strain D1701 is highly attenuated as a result of serial cell culture passage during which it underwent genomic rearrangements including an enlargement of the inverted terminal repeats to approximately 20 kbp [40], [41]. As a consequence, open reading frames (ORFs) 120 to 134 are diploid in this strain, whereas only ORF 134 is diploid in strain NZ2. In addition, at least one ORF (005) was deleted during this rearrangement [41].

The significance of these and other differences between the strains with respect to immunomodulatory functions of iORFV remain unclear and await detailed comparisons of the two strains. Both strains D1701 and NZ2 did show significant antiviral activity against HBV in the HBV-transgenic mouse model. In this model, however, NZ2 was significantly more potent in both reducing HBV-DNA and HBcAg. Dosages were compared based on TCID_50_ of active virus. However, poxvirus preparations usually contain non-viable (non-plaque forming) particles. The ratio of these to plaque forming units varies among strains and according to the method of virus preparation or assay [42], [43]. In depth studies of this phenomenon in ORFV have not been published. However, in unpublished work we have confirmed that there are significant amounts of non-plaque forming virus in preparations of ORFV. We do not know the molecular basis of differences of iORFV D1701-mediated immunomodulation vs. that of NZ2, however, apparently iORFV NZ2 is able to induce IFN-γ expression in the mouse at higher levels than D1701 at comparable dose equivalents of active virus. This is consistent with our previous observation that anti-HBV activity of iORFV is mediated by its ability to induce IFN-γ expression [11]. We have recently identified ORFV multi-gene fragments containing 27 open reading frames with immunomodulatory activity using a vaccinia virus/ORFV expression library [44]. After further analyses, two proteins (ORFs 102 and 103) were expressed, purified and demonstrated immunostimulatory activity and antiviral activity *in vitro* and *in vivo*, respectively [44]. The amino acid sequences of ORFs 102 and 103 of strain D1701 are 93.8% and 95.5%, respectively, identical to the corresponding ORFs of strain NZ2. Ongoing and future studies such as a comparison of these proteins of various strains of ORFV may add to the understanding of the mechanisms involved in iORFV-mediated immunomodulatory and antiviral activity.

Immunomodulatory treatment with IFN-α is a standard of care component of HCV infection. As IFN-α is amongst the cytokines that are induced by iORFV we analyzed the potential of iORFV to inhibit HCV. Both iORFV strains were active in an *in vitro* HCV replication model. Although the data represent a proof of concept *in vitro,* they do not allow conclusions on differences in potency. Optimization of the analysis system might reveal such differences. We previously demonstrated that iORFV induced hepatic expression of IFN-γ and IL-10 in rats [12]. Furthermore, iORFV D1701 elicited anti-fibrotic activity in two models of liver fibrosis although the causative fibrogenic agent was not removed [12]. Our results described in this paper show that administration of iORFV, strain NZ2, reduces liver fibrosis in a dose-dependent fashion as well. Moreover, a dose of 5×10^5^ TCID_50_ NZ2 almost completely prevented the formation of fibrotic collagen while an approximately tenfold higher dose of D1701 was needed to reach a comparable effect in the same model [12]. IFN-γ elicits anti-fibrotic activity in hepatic stellate cells (HSC), which participate in matrix remodeling and deposition in liver fibrosis. The antifibrotic effect is mediated by the ability of IFN-γ to suppress the overexpression of TGFß mRNA, which is followed by the suppression of procollagen mRNAs [45]–[49]. We have also demonstrated that iORFV inhibits expression of collagen type 1 and IFN-γ in rat hepatic stellate cells *in vitro* [12]. Here, we show that transformation of hepatic stellate cells is inhibited by iORFV *in vivo*. The transformation of hepatic stellate cells into myofibroblasts is accompanied by the expression of α-smooth muscle actin (α-SMA) by transformed cells, induced by liver injury, is an important trigger for hepatic fibrogenesis. On the other hand, IL-10 has been described to possess potential anti-fibrotic activity [50], [51]. The hypothesis that iORFV may also act anti-inflammatory in the fibrosis models via induction of IL-10 is supported by functional liver tests which showed generally reduced transaminase-levels in ORFV-treated compared to placebo-treated animals exposed to CCL_4_ [12].

In summary, the data presented here demonstrate that iORFV can induce antiviral activity against two important human liver pathogens, HBV and HCV in preclinical models. Importantly, iORFV demonstrated anti-fibrotic activity in a model of liver fibrosis, which is a consequence of chronic infection with either HBV or HCV. Additional studies are necessary and warranted to further explore the underlying mechanisms that determine the combination of antiviral activity and the potentially complex liver-protective activity that is mediated by inactivated ORFV.
